# Deletion of classical transient receptor potential 1, 3 and 6 alters pulmonary vasoconstriction in chronic hypoxia-induced pulmonary hypertension in mice

**DOI:** 10.3389/fphys.2022.1080875

**Published:** 2022-12-07

**Authors:** Kathrin Malkmus, Monika Brosien, Fenja Knoepp, Lisa Schaffelhofer, Friedrich Grimminger, Christoph Rummel, Thomas Gudermann, Alexander Dietrich, Lutz Birnbaumer, Norbert Weissmann, Simone Kraut

**Affiliations:** ^1^ Cardiopulmonary Institute (CPI), Universities of Giessen and Marburg Lung Center (UGMLC), Member of the German Center for Lung Research (DZL), Justus-Liebig-University, Giessen, Germany; ^2^ Institute of Veterinary Physiology and Biochemistry, Justus-Liebig-University, Giessen, Germany; ^3^ Walther Straub Institute for Pharmacology and Toxicology, Member of the DZL, Ludwig Maximilians University, Munich, Germany; ^4^ Institute of Biomedical Research (BIOMED), Catholic University of Argentina, Buenos Aires, Argentina; ^5^ Laboratory of Signal Transduction, National Institute of Environmental Health Sciences (NIEHS), Durham, United States.

**Keywords:** chronic hypoxia-induced pulmonary hypertension, pulmonary hypoxic vasoconstriction, pulmonary vascular remodeling, pulmonary arterial smooth muscle cells (PASMCs), proliferation, TRPC, mice

## Abstract

Chronic hypoxia-induced pulmonary hypertension (CHPH) is a severe disease that is characterized by increased proliferation and migration of pulmonary arterial smooth muscle cells (PASMCs) leading to pulmonary vascular remodeling. The resulting increase in pulmonary vascular resistance (PVR) causes right ventricular hypertrophy and ultimately right heart failure. In addition, increased PVR can also be a consequence of hypoxic pulmonary vasoconstriction (HPV) under generalized hypoxia. Increased proliferation and migration of PASMCs are often associated with high intracellular Ca^2+^ concentration. Recent publications suggest that Ca^2+^-permeable nonselective classical transient receptor potential (TRPC) proteins—especially TRPC1 and 6—are crucially involved in acute and sustained hypoxic responses and the pathogenesis of CHPH. The aim of our study was to investigate whether the simultaneous deletion of TRPC proteins 1, 3 and 6 protects against CHPH-development and affects HPV in mice. We used a mouse model of chronic hypoxia as well as isolated, ventilated and perfused mouse lungs and PASMC cell cultures. Although right ventricular systolic pressure as well as echocardiographically assessed PVR and right ventricular wall thickness (RVWT) were lower in TRPC1, 3, 6-deficient mice, these changes were not related to a decreased degree of pulmonary vascular muscularization and a reduced proliferation of PASMCs. However, both acute and sustained HPV were almost absent in the TRPC1, 3, 6-deficient mice and their vasoconstrictor response upon KCl application was reduced. This was further validated by myographical experiments. Our data revealed that 1) TRPC1, 3, 6-deficient mice are partially protected against development of CHPH, 2) these changes may be caused by diminished HPV and not an altered pulmonary vascular remodeling.

## Introduction

Chronic hypoxia induced pulmonary hypertension (CHPH) is a serious disease that affects a wide range of people worldwide—with increasing yearly prevalence (Galiè et al., 2015; Hoeper et al., 2016; Wijeratne et al., 2018). However, the treatment options for patients suffering from CHPH are limited. Thus, further investigations—not only to understand the molecular pathways triggering the development of the disease more in detail, but also to find possible treatment options—are needed (Galiè et al., 2015; Wijeratne et al., 2018). In CHPH the mean pulmonary artery pressure is elevated above normal levels, and is suggested to be dependent on an increased muscularization of pulmonary arterial vessels. The subsequent increase in pulmonary vascular resistance (PVR) results in an increased afterload, the development of right ventricular hypertrophy and ultimately right ventricular failure and death (Han et al., 2007; Vonk Noordegraaf and Galiè, 2011; Ryan and Archer, 2014; Grimminger et al., 2017). In addition, hypoxic pulmonary vasoconstriction (HPV)—as an essential mechanism of the lung to match pulmonary blood perfusion and alveolar ventilation—can contribute to an elevated pulmonary vascular pressure. Under conditions of regional alveolar hypoxia, HPV maintains pulmonary gas exchange. However, if alveolar hypoxia is generalized, HPV induces vasoconstriction of all precapillary vessels of the lung and thus, induces an increase in pulmonary vascular pressure/PVR (Sommer et al., 2020).

Vascular remodeling in CHPH largely affects the tunica media, which is primarily composed of pulmonary arterial smooth muscle cells (PASMCs) (Yu et al., 2004; Stenmark and McMurtry, 2005; Tuder et al., 2007; Barman et al., 2019). Elevation in PASMC-proliferation increases the muscularization of small pulmonary arteries (Schermuly et al., 2011; Fernandez et al., 2015; Stenmark et al., 2018). In addition to proliferation, an increased migration of PASMCs is presumed to contribute to the muscularization of previously non-muscularized vessels (Stenmark et al., 2018) and thus, further aggravates the disease.

A change in the intracellular Ca^2+^concentration ([Ca^2+^]_i_) is hypothesized to be one of the reasons for the altered PASMC phenotype as increased [Ca^2+^]_i_ is crucial for proliferation, migration and apoptosis of PASMCs and therefore, highly involved in vascular remodeling (Pietra et al., 2004; Stenmark et al., 2009; Fernandez et al., 2015; Stenmark et al., 2018).

Amongst others [Ca^2+^]_i_ is regulated *via* classical transient receptor potential (TRPC) proteins, which are non-specific cation channels permeable for both Ca^2+^ and Na^+^. As an increase in Na^+^ activates voltage-gated Ca^2+^ channels, which also increases cytosolic Ca^2+^ (Gudermann et al., 2004), Ca^2+^ conductivity mainly contributes to the crucial role of TRPC proteins in CHPH development (Malczyk et al., 2013; Xia et al., 2014; Smith et al., 2016). Previous investigations showed that an upregulation for TRPC3 and 6 in human patients suffering from IPAH (Yu et al., 2004) as well as for TRPC1 and 6 in mice exposed to chronic hypoxia (Malczyk et al., 2013; Xia et al., 2014). In addition, it was shown in mice, that CHPH was less severe when TRPC1 was deleted (Malczyk et al., 2013) while TRPC6 seems to be crucial for acute HPV (Weissmann et al., 2006). Of interest, deletion of a single TRPC channel was described to be compensated by a counter-regulation of other TRPC channels (Dietrich et al., 2005b). To overcome such difficulties, we here investigated mice with a simultaneous deletion of TRPC 1, 3 and 6 (TRPC1/3/6^−/−^) channels. We hypothesize that TRPC1/3/6^−/−^ mice would be protected against the development of CHPH. To answer this question, we performed investigations in chronically hypoxic mice, isolated, ventilated and perfused lungs and on the cellular level in PASMCs.

## Methods

### Animals, material and methods

All experiments were approved by the governmental authorities (RP Giessen, Hesse, Germany; Az: V 54–19 c 20 15 h 01 GI 20/10 No. G10/2017) in accordance with the German animal welfare law and the European legislation for the protection of animals used for scientific purposes (2010/63/EU). Group size estimation was based on previous experiments performed in our laboratory and calculated using Sigma Stat 3.5 (Systat Software Inc, San Jose, United States) with a power of 0.80 and an alpha of 0.05. All investigators were blinded whenever possible. Animals were kept under controlled conditions (10 h dark/14 h light cycle, water and food (Altromin, Lange, Germany; No. 1324) supply *ad libitum;* group housed (2–5 per cage whenever possible) at 22°C ± 2°C and 55% ± 10% humidity). Mice were handled at least for 3 days before start of experiments to accustom them to the experimental procedures.

### Animals, genotyping and experimental design

**FIGURE 1:**
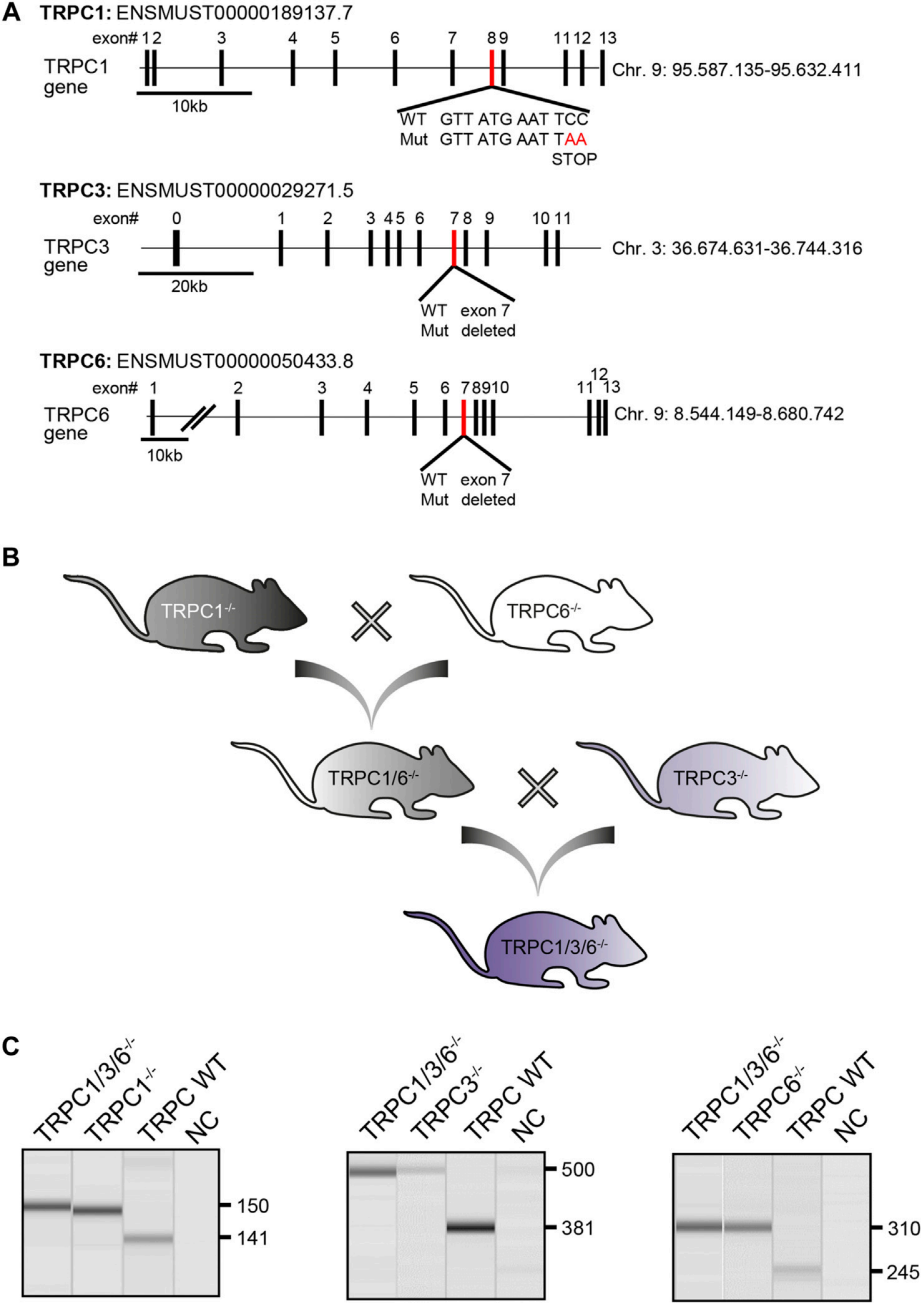
Generation and validation of TRPC1/3/6^−/−^ mice. **(A)** Schematic illustration depicting the targeted disruption of the murine *trpc1*, *trpc3* and *trpc6* gene. Exons are either represented as black (nontargeted exons) or red (targeted exons) boxes. Figure adapted from (Dietrich et al., 2005b; Dietrich et al., 2007; Hartmann et al., 2008) **(B)** Graphical illustration of the breeding scheme to generate TRPC1/3/6^−/−^ mice. **(C)** Representative gel-pictures of PCR products for genotyping. Genomic DNA was isolated from ear punches of TRPC1/3/6^−/−^, TRPC1^−/−^, TRPC3^−/−^ and TRPC6^−/−^ mice and used for PCR before being separated on an agarose gel. Left gel: Amplified WT (141 bp) and mutated (150 bp) *trpc1* product, middle: WT (381 bp) and mutated (500 bp) *trpc3* product and right: WT (245 bp) and mutated (310 bp) *trpc6* product. The first lane on each picture represents the TRPC1/3/6^−/−^ mice, the second lane the respective control knockout mouse, the third lane the WT mouse and the forth lane the negative control (NC). bp, base pairs.

Adult TRPC WT (B6.Cg-*Trpc*1^*+*^ *Trpc*3^*+*^ *TRPC*6^*+*^/) and TRPC1/3/6^−/−^ (B6.Cg-*Trpc*1^*tm1Lbi*^ *Trpc*3^*tm1.1Akon*^ *Trpc*6^*tm1Lbi*^/) mice of either sex and at an age of 12–16 weeks were used in this study. Mice were generated at the Walther Straub Institute for Pharmacology and Toxicology, Ludwig Maximilians University, Munich by breeding TRPC1^−/−^ (B6.Cg-Trpc1^tm1Lbi^) (Dietrich et al., 2007) and TRPC6^−/−^ (B6.Cg-TRPC6^tm1.Lbi^) mice (Dietrich et al., 2005b) to generate TRPC1/6^−/−^ double knockout mice (B6.Cg-TRPC1^tm1.Lbi^-TRPC6^tm1.Lbi^). By crossbreeding TRPC1/6^−/−^ with TRPC3^−/−^ (B6.Cg-TRPC6^tm1.Lbi^) mice (Hartmann et al., 2008), we generated TRPC1/3/6^−/−^ mice. An illustration of the breeding scheme can be found in Figure 1B. The genotyping of TRPC1/3/6^−/−^ mice was performed by analyzing *trpc*1, *trpc*3 and *trpc*6 separately. Genomic DNA was extracted using the Hotshot method. Briefly, ear punches of mice were dissolved in NaOH (50 mM) and incubated at 95°C for 15 min, vortexed and incubated in Tris solution (1 M, pH 8.0). The extracted genomic DNA was mixed with genotyping solution using GoTaq^®^ G2 Flexi DNA Polymerase Kit (Promega, #M7805) and Primers (0.1–0.2 µM) and followed by PCR. Afterwards, the PCR products were separated on a 1.5% sodium borate agarose gel (120 V, 120 mA, 30–45 min) containing Sybr safe DNA gel stain (BioRad, #1725124) using 6x DNA loading dye (ThermoFisher Scientific, Waltham, Massachusetts, United States) and visualized using the QIAxcel Advanced System (Qiagen, Hilden, Germany). For *trpc*1 the following primers were used: C1-F1: 5′- TCT ATG GCT TCT GAG GCG GA, C1-R1: 5′- GCA TTA TTA ATA TCT GAG TCA TTT TCT TAT TGG CAA AAT GAG G, C1-F2: 5′ GGC AAC CTT TGC CCT CAA AGT GGT GGC, C1-R2: 5′- AGT GAA TAT ATA TAT ATC AGA CAT AGA TTT GGG. Predicted PCR products were 141 bp for WT and 150 bp for the mutated *trpc*1 PCR product. For *trpc*3 the following primers were used: C3-F: 5′-AAA GCT CTG GTT GCT CTT GC, C3-R1: 5′ CCA AAA TGC ACA TAG AAG CTA A and C3-R2: 5′-CAT ATT TGA GGA CAA CAG AAG TCA C. Predicted reaction products were 381 bp for WT and 500 bp for the mutated *trpc*3. For *trpc*6 the following primers were used: C6-F1: 5′ GGG TTT AAT GTC TGT ATC ACT AAA GCC TCC, C6-R1: 5′ ACG AGA CTA GTG AGA CGT GCT ACT TCC, C6-F2: 5′ CAG ATC ATC TCT GAA GGT CTT TAT GC, C6-R2: 5′ CAT CAG GAC CCC GAG CAC CAC ATA C. Predicted PCR products were 245 bp for WT and 310 bp for the mutated *trpc*6. Information on RT-PCR analysis in aortas, brain Purkinje or lung cells of the respective single knockout mice have been previously described (Dietrich et al., 2005a; Dietrich et al., 2007; Hartmann et al., 2008).

For the characterization of the PH phenotype, mice (n = 15 per group, except for the hypoxia-treated TRPC1/3/6^−/−^ mice n = 14) were randomized and exposed either to normobaric normoxia or hypoxia (10% O_2_) for 28 days using a ventilated chamber (Biospherix, Parish, NY, United States) as described previously (Sommer et al., 2017). Afterwards, all mice underwent echocardiography followed by hemodynamic measurements and organ harvesting. For *in vivo* measurements numbers for final analysis vary (normoxia: TRPC WT n = 9–11 and TRPC1/3/6^−/−^ n = 11–14; hypoxia: TRPC WT n = 13–14 and TRPC1/3/6^−/−^ n = 13–14) due to technical reasons, e.g., placement of the catheter or echocardiographic transducer.

### Echocardiography, *in vivo* hemodynamics and blood gas analyses

All methods were performed as previously described (Veith et al., 2022). For anesthesia, 1.5–3% isoflurane gas in room air supplemented with 100% O_2_ (flow rate: 1 l/min) was used. Transthoracic echocardiography was performed using a VEVO2100 system (Visualsonics, Toronto, Canada RRID: SCR_015816) equipped with a 40 MHz transducer. To ensure physiological conditions, mice were placed on a thermoregulating plate that was connected to a rectal thermometer (to monitor body temperature) as well as electrocardiogram electrodes to observe heart rate. To obtain high-resolution images, mice were shaved followed by application of a pre-warmed ultrasound gel. Tricuspid annular plane systolic excursion (TAPSE) was measured in the apical four-chamber view and right ventricular wall thickness (RVWT) in a modified parasternal short axis view. Cardiac index (CI) was calculated as the product of the velocity-time integral (VTI) of the pulsed-Doppler tracing in the right ventricular outflow tract (RVOT), the cross-sectional area of the RVOT and the heart rate (HR) divided by body weight (BW) using the following formula: CI=((RVOTxVTI)xHR)/BW. Pulmonary vascular resistance (PVR) was calculated using the following formula: PVR=(80/3)×RVSP/cardiac output as previously described (Pullamsetti et al., 2017). For hemodynamic measurements, mice were tracheotomized and connected to a small animal ventilator MiniVent type 845 (Hugo Sachs Elektronik, March-Hugstetten, Germany). Then, a Millar catheter (Model SPR-671 Pressure Catheter; Millar Instruments, Inc, Houston, United States) was advanced into the right heart *via* access through the jugular vein and the right ventricular systolic pressure (RVSP) was recorded using the PowerLab system and LabChart 7.0 software (ADInstruments GmbH, Spechbach, Germany; RRID: SCR_001620). Subsequently, the catheter was inserted into the right carotid artery and systemic arterial pressure (SAP) was recorded. After data acquisition mice were exsanguinated under deep anesthesia (5% isoflurane with O_2_ supplement). Blood was collected *via* a capillary blood collection tube to measure hematocrit.

### Lung fixation, organ harvest, and right ventricular hypertrophy assessment

Lung fixation and organ harvest were performed as previously described (Veith et al., 2022). Briefly, a catheter was inserted into the pulmonary artery, and afterwards lungs were flushed with saline (Otto Fischer GmbH, Saarbrücken, Germany) under a constant pressure of 22 cm H_2_O for 5 min to prevent collapsing of the vessels. Subsequently the left lung was removed and incubated in 3.5% neutral buffered formalin (Otto Fischer GmbH) overnight at room temperature, followed by 24 h washing in PBS at 4°C. Finally, the fixed lung lobes were dehydrated in ethanol (Otto Fischer GmbH) and embedded in paraffin followed by histological investigations. Right ventricular hypertrophy was quantified by assessment of the Fulton Index and calculated as follows: right ventricular weight/left ventricular plus septal weight.

### Vascular morphometry

To quantify the degree of pulmonary vessel muscularization, 3 µm sections of paraffin-embedded lungs were stained with antibodies against α-smooth muscle actin (1:700 dilution; clone 1A4, Sigma-Aldrich, Munich, Germany, Cat# A2547, RRID:AB_476701, antibody validated by manufacturer ) and von Willebrand-factor (1:2000 dilution; Dako, Glastrup, Denmark, Agilent Cat# A0082, RRID:AB_2315602, antibody validated by manufacturer) as described previously (Dumitrascu et al., 2006). Pulmonary vessels with a diameter of 20–70 µm were analyzed from each lung using the Leica Qwin Software (Leica, Wetzlar, Germany). Lung sections were analyzed by meandering the slides at a magnification of × 400 and including every second row into the calculation (at least 100 vessels). Quantification was done by determining the percentage of α-smooth muscle actin-positive areas of the tunica media of all counted vessels for every mouse and group (≙ mean muscularization).

### Primary cell cultures

Murine microvascular PASMCs were isolated and cultured as described previously (Malczyk et al., 2013). Briefly, lungs were filled with 0.5% low-melting-point agarose (type VII, Sigma-Aldrich, #A9414) and 0.5% Fe_3_O_4_ (Sigma-Aldrich, #518158), mechanically shredded using scissors and digested with collagenase (80 U/ml, Sigma-Aldrich, #C0773) for 1 h. Then, tissue was washed in PBS (Capricorn Scientific, Ebsdorfergrund, Germany, #PBS-2A) using a magnet and PASMCs attached to microvascular pulmonary vessels were seeded using Smooth Muscle Basal Medium 2 (PromoCell, Madison, Wisconsin, United States, Cat# C-22262) containing 5% Smooth Muscle Basal Medium 2 Supplement (PromoCell, Cat# C-39267), 0.5% Normocin (Invivo-Gen, Toulouse, France) and 10% FCS (Sigma-Aldrich, St. Louis, Missouri, United States, Cat# F0804). It takes several hours up to 1 day until the PASMCs start outgrowing from the isolated precapillary pulmonary vessel pieces. During that time, the cells are already adherent. After 2–3 days, PASMCs are sufficiently grown to be used in experiments. Growing conditions can be normoxic or hypoxic from day 0.

### Proliferation assay

PASMC proliferation was assessed using the Click-iT Edu Alexa Fluor 488 Imaging Kit (ThermoFisher Scientific, Waltham, Massachusetts, United States, Cat#10337). Isolated PASMCs (passage 0) cultured in 24-well plates were exposed to chronic hypoxia for 96 h (1% O_2_, 5% CO_2_, balanced with N_2_), incubated with 5-Ethynyl-2′-Desoxyuridin (EdU, 10 µM) for further 24 h under chronic hypoxia and finally fixed with acetone-methanol (1:1; Sigma-Aldrich, St. Louis, Missouri, United States, Cat#32201 and Cat#32213) for 5 min at room temperature. Fixed PASMCs were blocked with 3% BSA (Sigma-Aldrich, St. Louis, Missouri, United States, Cat#A7030) and stained according to manufacturer’s protocol. Afterwards, PASMC nuclei were visualized with Hoechst^®^33342 (4µM; ThermoFisher Scientific, Cat# 62249). Fluorescence emission was detected using Leica DMI6000 CS fluorescence microscope (Leica, Wetzlar, Germany). Hoechst-positive (Hoechst^+^; total cell number) and EdU-positive (EdU^+^; proliferation cell number) PASMCs were counted using the Leica Las X (Leica, Wetzlar, Germany) software. Finally, proliferation rate was calculated by assessing the ratio between EdU^+^ and Hoechst^+^ PASMCs as previously described (Malczyk et al., 2013).

### Apoptosis assay

Apoptosis of PASMCs was assessed using the Alexa Fluor 488 Click-iT™ Plus TUNEL Assay (ThermoFisher Scientific, Cat# C10617). Isolated PASMCs were cultured in 96-well plates (10,000 PASMCs/cm^2^, passage 1) under hypoxic conditions (1% O_2_, 5% CO_2_, balanced with N_2_), for 72 h. Afterwards, PASMCs were fixed with acetone-methanol (1:1) for 5 min at room temperature before apoptotic cells were stained according to manufacturer’s protocol. Finally, cell nuclei were visualized by using Hoechst^®^33342 (4 µM; ThermoFisher Scientific, Cat# 62249). Hoechst^+^ (total cell number) and TUNEL-positive (TUNEL^+^) PASMCs were counted by using Leica DMI6000 CS fluorescence microscope and Las X software (Leica, Wetzlar, Germany). Apoptosis was estimated by calculating the ratio between TUNEL^+^ and Hoechst^+^ PASMCs and normalized by daily average.

### Migration assay

Migration of PASMCs was analyzed by measuring the transwell emigration rate using a Incucyte^®^ Clearview 96-well plate for chemotaxis (Sartorius, Goettingen, Germany, Cat# 4582). Cells were exposed to chronic hypoxia (1% O_2_, 5% CO_2_, balanced with N_2_) for 72 h and seeded on the upper membrane of an Incucyte^®^ Clearview 96-well plate (20,000 PASMCs/cm^2^, passage 1) in hypoxic Smooth Muscle Basal Medium 2 containing 0.5% Normocin (Invivo-Gen, Toulouse, France). To enable migration, the lower reservoir was filled with hypoxic Smooth Muscle Basal Medium 2 containing 0.5% Normocin and 20% FCS. Afterwards, PASMCs were analyzed using an Incucyte ZOOM Live-Cell Analysis System (Sartorius) under chronic hypoxia. Pictures of the upper and lower side of the membrane were taken hourly over a 48 h period. Migration rate was evaluated using the Incucyte^®^ ZOOM software (Sartorius) by counting the number of migrated PASMCs.

### Western blot

For the quantification of proteins in PASMCs, western blots were performed as previously described (Veith et al., 2022). The following antibodies were used: mouse monoclonal anti-β-actin (1:50,000; Sigma-Aldrich, Cat# A5316; RRID:AB_476743), rabbit polyclonal anti-p38 mitogen-activated protein kinase (MAPK) (1:1000; Cell Signalling Technology, Danvers, MA, United States, Cat# 9212, RRID:AB_330713, antibody was validated by Sato et al. (Sato et al., 2014)), rabbit monoclonal anti-phospho-p38 MAPK (1:1000; Cell Signalling Technology, Danvers, MA, United States, Cat# 4511, RRID:AB 2139682), goat anti-mouse horseradish peroxidase (HRP) conjugate (1:5000; Promega, Mannheim, Germany, Cat# W402B, antibody was validated by Sato et al. (Sato et al., 2014)) and goat anti-rabbit HRP conjugate (1:5000; Promega, Cat# W401B). Logarithmic values of the data were used for the statistical analysis.

### Isolated, ventilated and perfused lung experiments

To analyze HPV in TRPC WT and TRPC1/3/6^−/−^ mouse lungs, isolated, ventilated and perfused lung experiments were performed as described previously (Sommer et al., 2017). Additional experiments for vasoreactivity were conducted by applying either the thromboxane A_2_ mimeticum 9,11-Dideoxy-9α,11α-methanoepoxyprostaglandin F2α (U46619, 33.3 nmol/l, Biomol, Hamburg, Germany, Cat# Cay16450-10) or KCl (11.25 mmol/l, Carl Roth GmbH, Karlsruhe, Germany, Cat# P017.3) after the acute hypoxic maneuver (10% O_2_, 5.3% CO_2_, balanced with N_2_).

### Measurements of vessel contractility and stiffness *via* wire myography

To assess contractility and stiffness of pulmonary arteries from TRPC WT and TRPC1/3/6^−/−^ mice, the left and right pulmonary artery after the first bifurcation were isolated. For aortic contractility measurements aortas from both genotypes were isolated. Directly after isolation, the vessels were mounted into a myograph (Multimyograph 630 M; Danish MyoTechnology A/S, Arhus, Denmark) and equilibrated in a physiological salt solution (PSS in mM: NaCl 119, KCl 4.7, CaCl_2_ 2.5, MgSO_4_ 1.17, KH_2_PO_4_ 1.18, EDTA 0.03, Glucose 5.5, NaHCO_3_ 25, pH 7.4) that was permanently gassed with a premixed gas-mixture of 21% O_2_, 5.3% CO_2_ , rest N_2_. After slowly raising the temperature to 37°C over 20 min, the vessels were normalized to a pre-tension of 2.66 kPa. To confirm the viability of the vessels and to assess the contractile response, the arteries were exposed to potassium-enriched PSS (KPSS in mM: NaCl 64.86, KCl 58.82, CaCl_2_ 2.5, MgSO_4_ 1.17, KH_2_PO_4_ 1.18, EGTA 0.03, Glucose 5.5, NaHCO_3_ 25, pH 7.4) for three times, separated by wash-out steps with PSS (3–5 min each). The third response to KPSS was used for analyzing contractility. Afterwards, the arteries were flushed with PSS until the baseline was reached, and then equilibrated for 30 min in Ca^2+^-free PSS (in mM, NaCl 119, KCl 4.7, MgSO_4_ 1.17, KH_2_PO_4_ 1.18, MnCl_2_ 2.5, EGTA 10, EDTA 0.03, Glucose 5.5, NaHCO_3_ 25, pH 7.4). The arterial stiffness was assessed as previously described (Gierhardt et al., 2022): The distance between the wires was increased stepwise while recording the corresponding force in mN. Using the DMT normalization feature in Labchart (ADInstruments, Dunedin, New Zealand), the resulting stress-strain curve displaying the relationship between the logarithmically transformed resting wall tension and the corresponding internal diameter was calculated *via* regression analysis. The tangential slope of the stress–strain curve was then determined as stiffness-parameter and is given in mN/mm.

### mRNA analysis by PCR array/quantitative real-time PCR (qRT-PCR)

Procedures for mRNA extraction, pre-amplification, cDNA synthesis, and PCR array/qRT-PCR were performed as described previously (Seimetz et al., 2011). The intron-spanning primers were designed by using the NCBI database and were as follows:

*B*_*2*_ *m* mouse*_*F: 5‘AGC​CCA​AGA​CCG​TCT​ACT​GG-3‘, *B*_*2*_ *m* mouse*_*R: 5‘-TTC​TTT​CTG​CGT​GCA​TAA​ATT​G-3‘

*Trpc*4 mouse_F: 5‘-GG​CGG​CGT​GCT​GCT​GAT-3‘,

*Trpc*4 mouse_R: 5‘-CCG​CGT​TGG​CTG​ACT​GTA​TTG​TAG-3‘

*Trpc*5 mouse_F: 5‘-AGT​CGC​TCT​TCT​GGT​CTG​TCT​TT-3‘,

*Trpc*5 mouse_R: 5‘-TTT​GGG​GCT​GGG​AAT​AAT​GG-3‘

*Trpc*7 mouse_F: 5‘-GTG​GGC​GTG​CTG​GAC​CTG-3‘, *Trpc*7 mouse_R: 5‘-AGA​CTG​TTG​CCG​TAA​GCC​TGA​GAG-3‘

### Data and analysis

All data are given as mean ± SEM. Differences between the groups were either assessed by analysis of variance (two-way ANOVA, comparison between 4 groups) or unpaired student’s t-test (comparison between 2 groups) after testing for normal distribution. Following the ANOVA, Sidak’s post-hoc test for multiple comparisons (GraphPad PRISM Version 9; GraphPad Software Inc. La Jolla, United States) was applied. For western blot analyses the logarithms of the measured values were used. Differences with a *p* value < 0.05 were considered statistically significant. All oxygen concentrations stated in the manuscript refer to sea level altitude.

## Results

### Simultaneous deletion of TRPC1, 3 and 6 partially protects against CHPH and RVH

**FIGURE 2:**
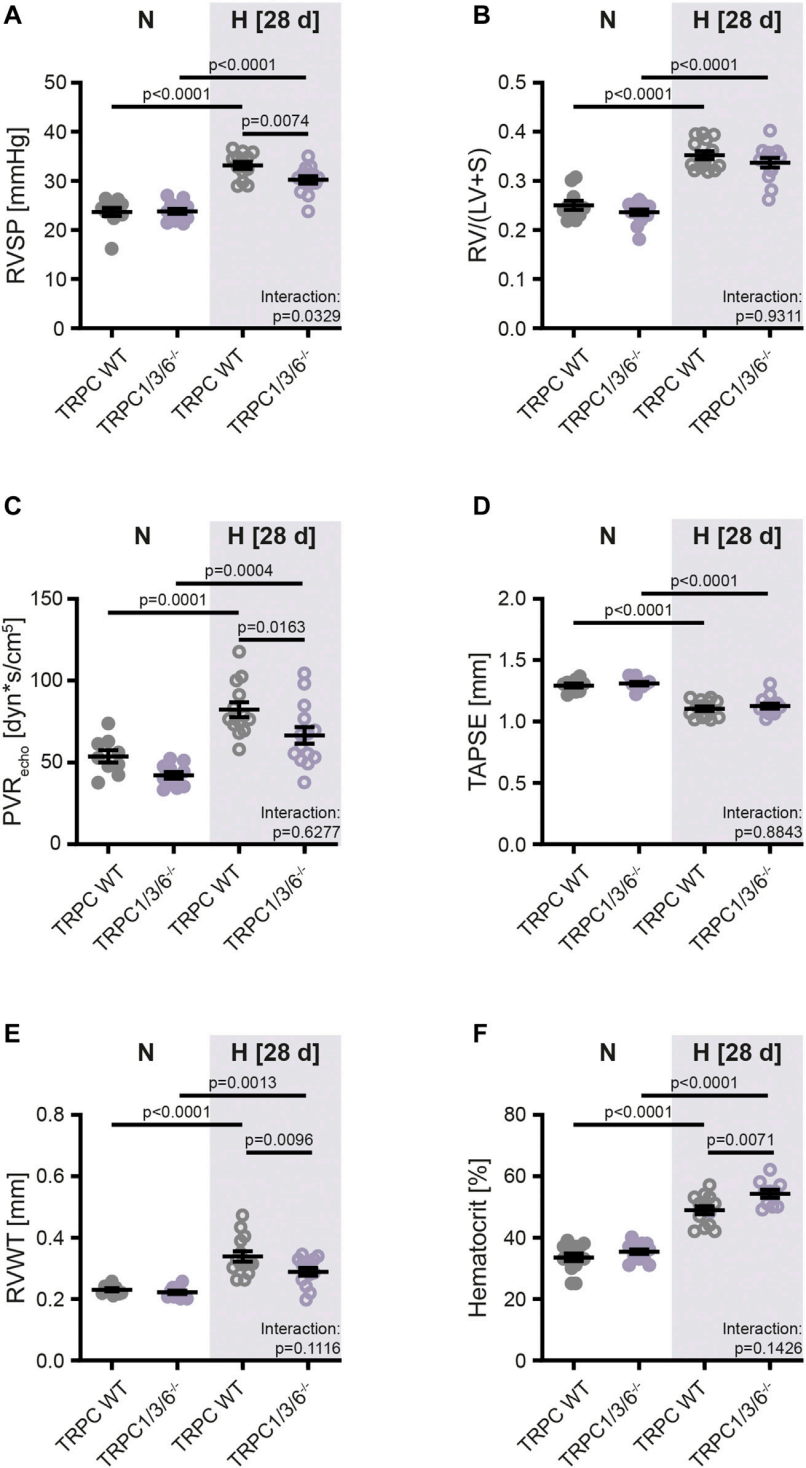
Development of pulmonary hypertension in TRPC WT and TRPC1/3/6^−/−^ mice upon chronic hypoxia. Mice were either exposed to normoxia (N: TRPC WT *n* = 15; TRPC1/3/6^−/−^ *n* = 15) or hypoxia (H: TRPC WT *n* = 15; TRPC1/3/6^−/−^ *n* = 14) for 28 days. **(A)** Right ventricular systolic pressure (RVSP; N: TRPC WT n = 11; TRPC1/3/6^−/−^ *n* = 13; H: TRPC WT *n* = 13; TRPC1/3/6^−/−^ *n* = 14). **(B)** Fulton Index (=ratio of right ventricular to left ventricular and septum mass (RV/(LV + S); N: TRPC WT *n* = 11; TRPC1/3/6^−/−^ *n* = 15; H: TRPC WT *n* = 14; TRPC1/3/6^−/−^ *n* = 14). **(C)** Echocardiographically assessed pulmonary vascular resistance (PVR_echo_; N: TRPC WT *n* = 9; TRPC1/3/6^−/−^ n = 11; H: TRPC WT n = 13; TRPC1/3/6^−/−^ n = 14). **(D)** Tricuspid annular plane systolic excursion (TAPSE; N: TRPC WT *n* = 9; TRPC1/3/6^−/−^ *n* = 10; H: TRPC WT *n* = 14; TRPC1/3/6^−/−^ *n* = 14). **(E)** Right ventricular wall thickness (RVWT; N: TRPC WT n = 9; TRPC1/3/6^−/−^ *n* = 11; H: TRPC WT *n* = 14; TRPC1/3/6^−/−^ *n* = 13). **(F)** Hematocrit (N: TRPC WT n = 14; TRPC1/3/6^−/−^ *n* = 14; H: TRPC WT *n* = 13; TRPC1/3/6^−/−^ *n* = 10). A significant interaction describes an interdependency between changes in genotype and exposure (normoxia-hypoxia). Significance was calculated using two-way ANOVA with Sidak post-hoc test.

To analyze the effects of TRPC1, 3 and 6 on development of PH, we have successfully generated TRPC1/3/6^−/−^ mice (Figures 1A–C). Exposure to chronic hypoxia for 4 weeks resulted in development of PH as assessed by measurement of RVSP (Figure 2A, Supplementary Figure S1A), Fulton Index (Figure 2B), echocardiographically determined PVR (Figure 2C), TAPSE (Figure 2D) as well as RVWT (Figure 2E, Supplementary Figure S1B) in both TRPC WT and TRPC1/3/6^−/−^ mice. Of interest, the hypoxia-induced increase in the RVSP, PVR and RVWT was less pronounced in the TRPC1/3/6^−/−^ mice, whereas their hematocrit was significantly higher when compared to the hypoxic TRPC WT mice (Figures 2A,C,E,F, Supplementary Figures S1A, B). By investigating TRPC1, 3 and 6-dependent effects on the systemic circulation we found a reduced vasoconstriction upon KCl application in myography experiments in isolated aortas (Supplementary Figure S2A), whereas systemic blood pressure was increased in both normoxia- and hypoxia-exposed TRPC1/3/6^−/−^ mice (Supplementary Figure S2B). Moreover, we detected a difference in heart rate between normoxic TRPC WT and TRPC1/3/6^−/−^ mice that was absent after exposure to chronic hypoxia (Supplementary Figure S2C) and accompanied by an unchanged CI (Supplementary Figure S2D). No obvious abnormalities regarding body weight (Supplementary Figure S2E), breeding performance, general health or neurology were observed.

### The partial protection against CHPH in TRPC1/3/6^−/−^ mice is not driven by an altered vascular remodeling

**FIGURE 3:**
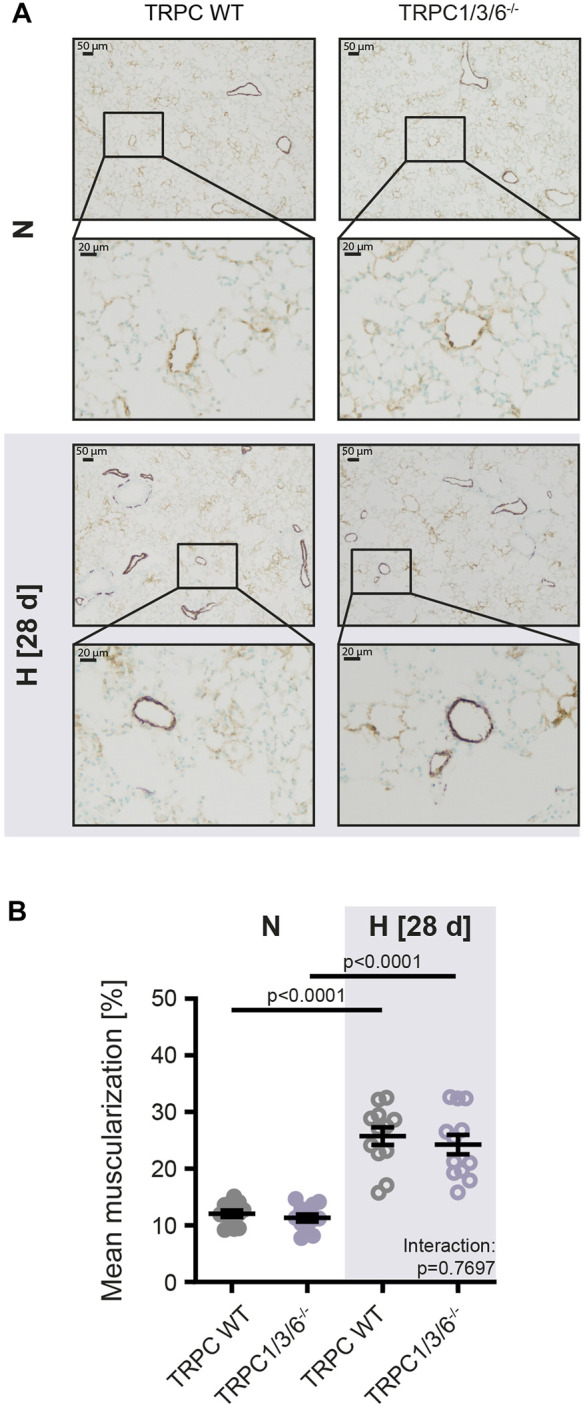
Vascular remodeling in TRPC WT and TRPC1/3/6^−/−^ mice upon chronic hypoxia. Mice were either exposed to normoxia (N: TRPC WT *n* = 15; TRPC1/3/6^−/−^ *n* = 15) or hypoxia (H: TRPC WT *n* = 15; TRPC1/3/6^−/−^ *n* = 14) for 28 days. **(A)** Representative histological images of lungs from all groups stained against α-smooth muscle actin (violet) and von Willebrand-factor (brown) and **(B)** mean muscularization of small pulmonary vessels (20–70 μm; *n* = 12 each group). Significance was calculated using two-way ANOVA with Sidak post-hoc test.

**FIGURE 4:**
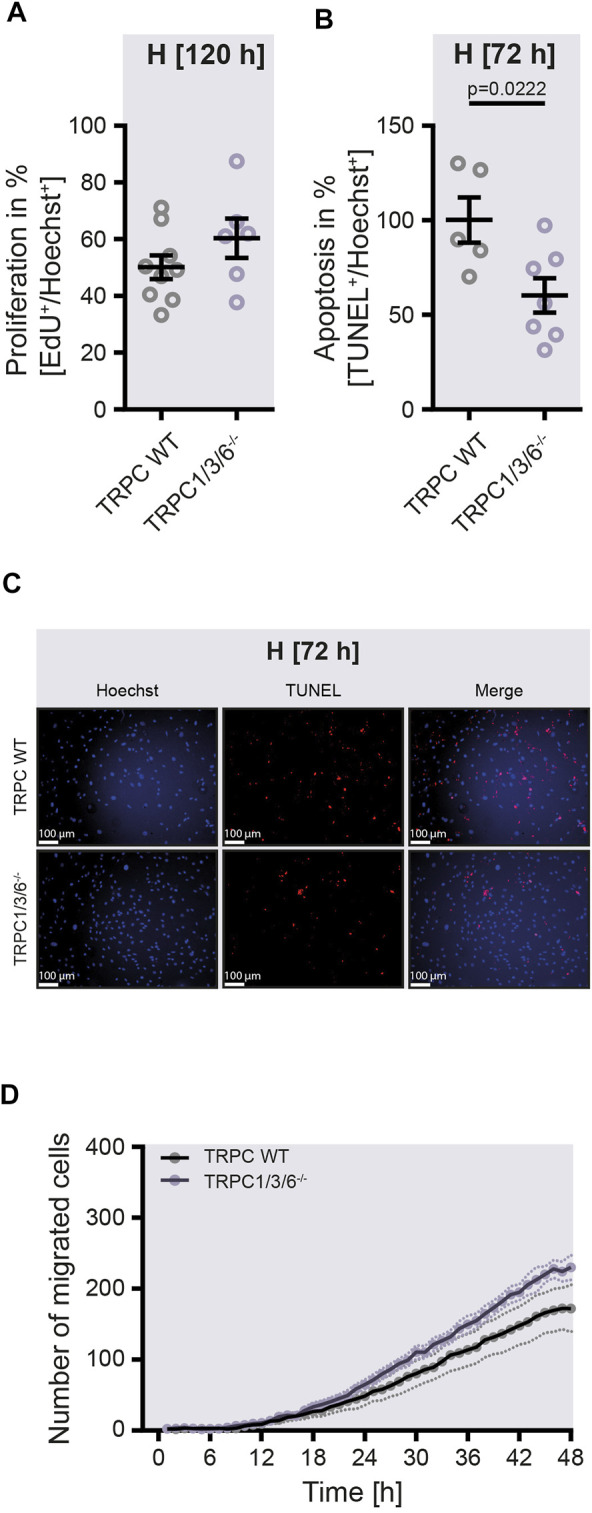
Proliferation, apoptosis and migration in isolated pulmonary arterial smooth muscle cells upon hypoxia. Pulmonary arterial smooth muscle cells (PASMCs) were either isolated from TRPC WT or TRPC1/3/6^−/−^ mice and exposed to hypoxia for 120 h **(A,D)** or 72 h **(B,C)**. **(A)** Proliferation was calculated by assessing the ratio between EdU^+^ and Hoechst^+^ PASMCs (TRPC WT *n* = 9; TRPC1/3/6^−/−^ *n* = 6). **(B)** Apoptosis was estimated by calculating the ratio between TUNEL^+^ and Hoechst^+^ PASMCs and normalized by daily average (TRPC WT *n* = 5; TRPC1/3/6^−/−^ *n* = 7). **(C)** Representative images of Hoechst^+^ or TUNEL^+^ PASMCs. **(D)** Migration was evaluated by counting the number of migrated PASMCs and recorded over the last 48 h of hypoxic exposure (TRPC WT *n* = 5; TRPC1/3/6^−/−^ *n* = 5). Significance was calculated using unpaired student’s *t*-test.

**FIGURE 5:**
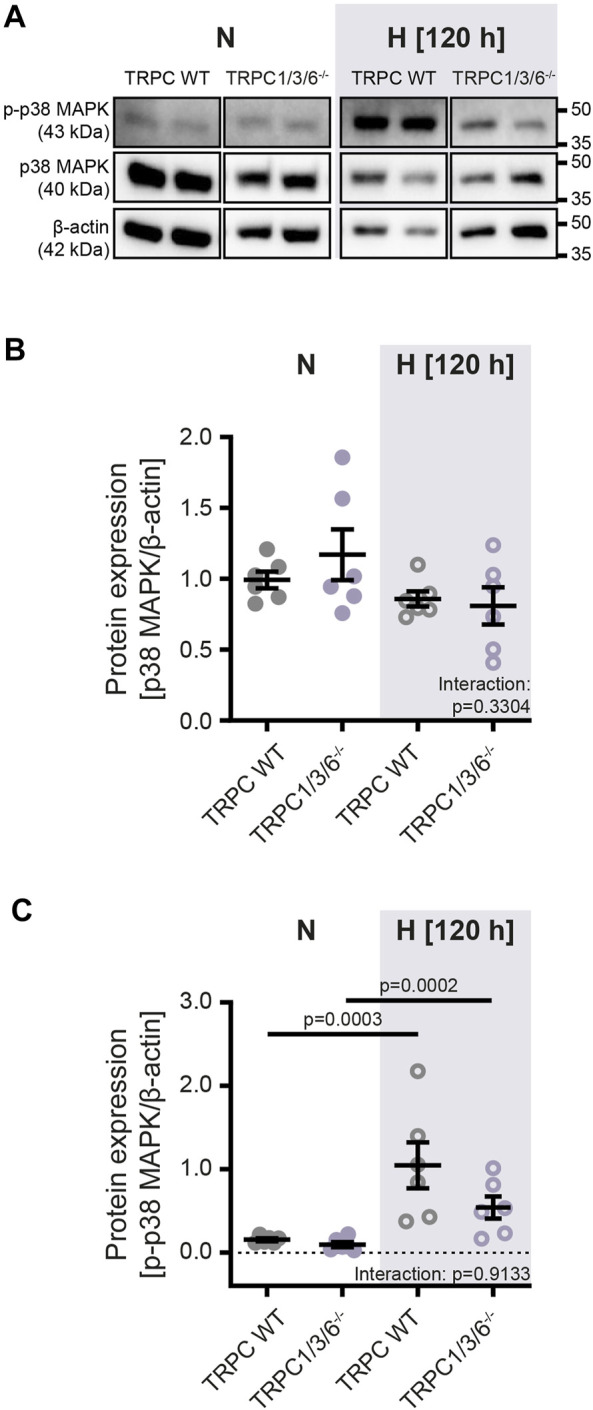
Protein expression of p38 MAPK and phospho-p38 MAPK in isolated pulmonary arterial smooth muscle cells after normoxic and hypoxic incubation. Pulmonary arterial smooth muscle cells were either isolated from TRPC WT or TRPC1/3/6^−/−^ mice and exposed to hypoxia or normoxia for 120 h. **(A)** Representative blot and densitometric analysis of **(B)** mitogen activated protein kinase p38 (p38 MAPK; 40 kDa) and **(C)** phospho-p38 (p-p38 MAPK; 43 kDa). Data were normalized to β-actin (42 kDa), *n* = 6 for each group. Significance was calculated using two-way ANOVA with Sidak post-hoc test.

To investigate whether the observed echocardiographic and hemodynamic differences between TRPC WT and TRPC1/3/6^−/−^ mice after chronic hypoxia are driven by pulmonary vascular remodeling, we performed a histological analysis of the degree of muscularization (Figures 3A,B). After exposure to chronic hypoxia, an increased mean muscularization was detected in both genotypes. However, no difference was observed between chronically hypoxic TRPC1/3/6^−/−^ and TRPC WT mice. Similarly, neither the proliferation, nor migration was different in isolated PASMCs after hypoxic incubation (Figures 4A,D), whereas apoptosis was decreased in the TRPC1/3/6^−/−^ mice (Figures 4B,C). Next we investigated p38 MAPK and its phosphorylation upon hypoxia in isolated PASMCs, as p38 MAPK has been reported to be involved centrally in abnormal proliferation of PASMCs (Li et al., 2020; Yue et al., 2020). Although phosphorylation of the p38 MAPK was increased upon hypoxic incubation in isolated PASMCs, again no differences could be observed between TRPC1/3/6^−/−^ and TRPC WT mice (Figure 5).

### Pulmonary vasoconstriction is attenuated in TRPC1/3/6^−/−^ mice

**FIGURE 6:**
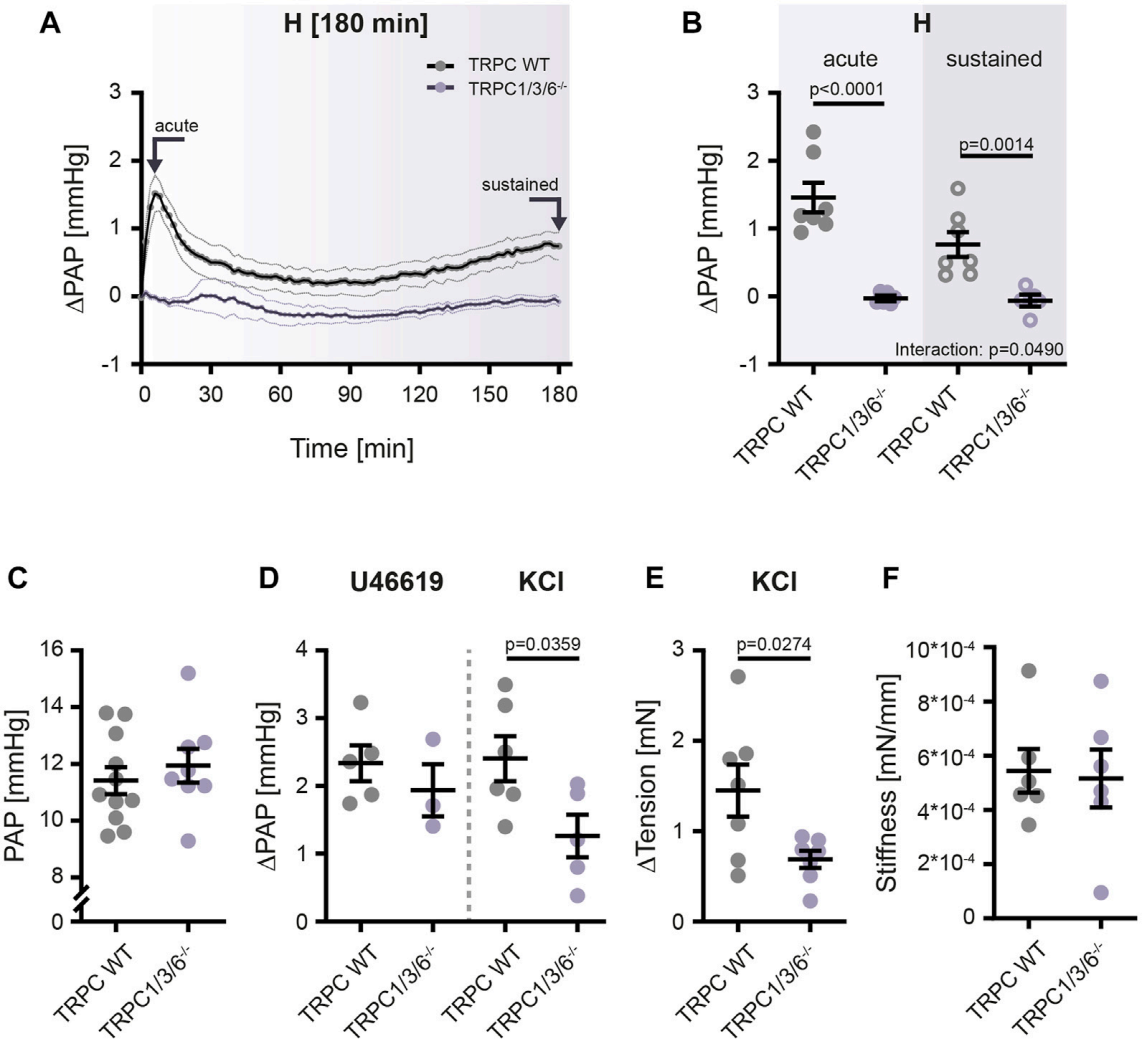
Hypoxia- and non-hypoxia-induced pulmonary vasoconstriction in TRPC WT and TRPC1/3/6^−/−^ mice. TRPC WT and TRPC1/3/6^−/−^ mice were investigated using the isolated, ventilated and perfused lung system for vasoreactivity to hypoxia and pharmacological stimulation. **(A)** Pulmonary arterial pressure (PAP) was recorded for 180 min and is given as mean ± SEM. Arrows indicate time points of data assessment shown in panel B. **(B)** Changes in PAP, related to the PAP directly prior to the onset of hypoxic ventilation (ΔPAP). Values for the acute as well as the sustained phase are plotted (TRPC WT *n* = 7; TRPC1/3/6^−/−^ *n* = 6). Significance was calculated using two-way ANOVA with Sidak post-hoc test. **(C)** Values for normoxic PAP directly prior to the onset of hypoxic ventilation and either application of the thromboxane A2 mimetic U46619 or KCl (TRPC WT n = 11; TRPC1/3/6^−/−^ *n* = 8). **(D)** Changes in PAP compared to PAP prior to application of U46619 or KCl under normoxic conditions (ΔPAP; U46619: TRPC WT *n* = 5; TRPC1/3/6^−/−^ *n* = 3; KCl; TRPC WT *n* = 6; TRPC1/3/6^−/−^ *n* = 5). **(E)** KCl-induced changes in the tension of isolated pulmonary arteries analyzed in wire myography experiments under normoxic conditions (TRPC WT *n* = 7; TRPC1/3/6^−/−^ *n* = 7). **(F)** Stiffness of isolated pulmonary arteries in TRPC WT and TRPC1/3/6^−/−^ mice. The stiffness is given as the tangential slope of the determined stress-strain curve (TRPC WT *n* = 6; TRPC1/3/6^−/−^ *n* = 6). A significant interaction describes an interdependency between changes in genotype and exposure (acute-sustained hypoxia). Significance was calculated using unpaired student’s *t*-test.

Since vascular remodeling induced by chronic hypoxia was not different between the two genotypes, we next investigated the strength of HPV and non-hypoxia-induced vasoconstriction in isolated, ventilated and perfused lungs (Figures 6A–D) as well as by wire myography in isolated pulmonary arteries (Figures 6E,F). The isolated lung experiments revealed that acute (peaking within 10 min) as well as sustained (observed over a time period of 180 min) HPV was almost absent in TRPC1/3/6^−/−^ mice as compared to TRPC WT mice (Figures 6A,B). Whereas both the normoxic pulmonary arterial pressure (Figure 6C) and the non-hypoxia-induced vasoconstriction elicited by the thromboxane mimetic U46619 was not different between TRPC1/3/6^−/−^ and TRPC WT mouse lungs, the vasoconstriction induced by application of KCl to the perfusate was reduced in TRPC1/3/6^−/−^ mice (Figure 6D). Similarly, reduced vasoconstriction upon KCl application in myography experiments was observed in isolated pulmonary arteries (Figure 6E). Pulmonary vascular stiffness was, however, unaltered between the two genotypes (Figure 6F).

### Impact of simultaneous deletion of TRPC1, 3 and 6 on other TRPC subfamily members

To further examine the altered –TRPC1, 3 and 6-dependent – functional response of PASMCs to the stimulus of exposure to chronic hypoxia, we performed expression analyses of other *Trpc* genes. TRPC2 channels were not investigated as they are mainly expressed and functional in the vomeronasal organ of mice, but not in the lung (Miller 2014). While *Trpc*4 expression revealed no differences between the two genotypes (Supplementary Figure S3A), PASMCs isolated from TRPC1/3/6^−/−^ mice showed significantly lower expression of *Trpc*5 in general (Supplementary Figure S3B). Moreover, the expression of *Trpc*7 in PASMCs was decreased in chronic hypoxia in TRPC WT mice but not in the TRPC1/3/6^−/−^ mice (Supplementary Figure S3C).

## Discussion

Our data demonstrate that 1) simultaneous deletion of TRPC1, 3 and 6 partially protects against CHPH despite an unchanged pulmonary vascular remodeling, and 2) this is associated with reduced pulmonary vasoconstriction possibly related to depolarizing stimuli only.

### Simultaneous deletion of TRPC1, 3 and 6 does not affect pulmonary vascular remodeling

The possible importance of either TRPC1, 3 or 6 alone was already described in the pathogenesis of PH. An upregulation of TRPC3 and TRPC6 was seen in PASMCs of patients suffering from IPAH (Yu et al., 2004). Moreover, an upregulation of TRPC1 in murine PASMCs was observed after exposure to chronic hypoxia (Malczyk et al., 2013). In relation to these results, previous studies revealed that a single deletion of TRPC1 in mice partially protects against the development of CHPH, without affecting HPV (Malczyk et al., 2013). In contrast, TRPC6 seems to be crucial for both acute HPV (Weissmann et al., 2006; Smith et al., 2015) and the development of CHPH (Xia et al., 2014). Additionally, a partial protection against CHPH was demonstrated for mice treated with BI-749327, a specific TRPC6 inhibitor (Jain et al., 2021). Of interest, Dietrich et al. (2005b) described a counter-regulation of TRPC3 in a single TRPC6^−/−^ mouse (Dietrich et al., 2005b). Such a compensatory counter-regulation might explain the only partial protection against development of CHPH. However, an investigation of the development of CHPH in mice by simultaneous deletion of TRPC1, 3 and 6 had not been performed. Examining TRPC1/3/6^−/−^ mice avoids the compensatory counter-regulation of TRPC3 as described in TRPC6 knockout mice (Dietrich et al., 2005b).

We found that TRPC1/3/6^−/−^ mice developed a lower degree of CHPH than TRPC WT mice, but most interestingly, this was not related to a decreased degree of pulmonary vascular muscularization. This finding was unexpected as the development of pulmonary hypertension is usually related to the degree of pulmonary vascular remodeling. However, not only vascular remodeling but also HPV determines pulmonary vascular resistance in hypoxia. Accordingly, our finding that acute and sustained HPV is almost absent in TRPC1/3/6^−/−^ mice might explain the reduced CHPH in the absence of altered muscularization of pulmonary arterial vessels.

In detail, our data revealed that TRPC1/3/6^−/−^ mice developed a lower degree of CHPH than TRPC WT mice after exposure to chronic hypoxia for 4 weeks as evident from a lower RVSP, RVWT and PVR_echo_. The fact that the echocardiographically assessed reduction in RVWT was not reflected by the Fulton Index suggests that echocardiography is a more sensitive parameter to evaluate RVH than weight determination. Moreover, we found a difference in heart rate between normoxic TRPC WT and TRPC1/3/6^−/−^ mice that was absent after exposure to hypoxia. Since CI was unchanged between the two genotypes, we assume that this is a general effect of anesthesia, which does not influence heart function.

In addition, we showed that hypoxia-exposed TRPC1/3/6^−/−^ mice developed a higher hematocrit than WT mice. Among other factors, a high hematocrit is described to be causative for the development of PH by elevating PVR and pulmonary arterial pressure due to increased blood viscosity (Hoffman 2011). Surprisingly, a different effect was found in EPO overexpressing mice that demonstrated a decreased PVR as well as a reduced vasoconstriction, despite a highly increased hematocrit (Weissmann et al., 2005). In this regard one might speculate that the increased hematocrit found in our TRPC1/3/6^−/−^ mice possibly contributes to the partial protection against CHPH.

Furthermore, the absence of an altered proliferation and migration of PASMC in chronic hypoxia is in line with the unaltered vascular remodeling between the two genotypes: Increased proliferation and migration as well as decreased apoptosis of PASMCs are crucial mechanisms for the development of vascular remodeling in CHPH (Pietra et al., 2004; Tajsic and Morrell, 2011; Stenmark et al., 2018). Moreover, it seems that a complex interaction between proliferation, migration and apoptosis is necessary to drive vascular remodeling, since our data revealed, that this pathomechanism is not affected by reduced apoptosis alone. Finally, the finding of the absence of a difference in proliferation between TRPC1/3/6^−/−^ and TRPC WT mice in our study is supported by the unaltered expression of p38 MAPK and its phosphorylation upon hypoxia in isolated PASMCs, as p38 MAPK has been reported to be involved centrally in abnormal proliferation of PASMCs (Li et al., 2020; Yue et al., 2020).

However, it should be noted that TRPC channels have previously been shown to be involved in proliferation, migration, apoptosis and pulmonary vascular remodeling in CHPH (Lin et al., 2004; Malczyk et al., 2013; Wang et al., 2013; Xia et al., 2014; Fernandez et al., 2015; Smith et al., 2015; Xu et al., 2021).

While in previous studies proliferation of PASMCs in TRPC1^−/−^ and TRPC1/6^−/−^ mice was reduced (Malczyk et al., 2013), contradictory findings investigating TRPC6 exist: although it seems that knockdown of TRPC6 in IPAH patients led to a reduced proliferation of PASMCs (Yu et al., 2004) TRPC6 is not involved in PASMC proliferation in mice (Malczyk et al., 2013). This last finding was confirmed by our TRPC1/3/6^−/−^ mice and might indicate that distinct TRPC proteins contribute differently to the development of PH by either different channel compositions or compensatory counter-regulations.

So far, data from human patients or mice that have investigated the role of TRPC3 in hypoxia-induced proliferation are not available. However, it was shown in PASMCs isolated from lipopolysaccharide treated rats, that proliferation induced by ET-1 after knockdown of TRPC3 was reduced (Jiang et al., 2016).

Another important pathomechanism driving vascular remodeling is migration. Here, only little is known about the effect of TRPC1, 3 and 6 on hypoxia-induced migration. However, previous studies investigating PASMCs isolated from IPAH patients demonstrated that activation of TRPC6 *via* calcium-sensitive receptors induces migration (Tang et al., 2016). Moreover, an upregulation of TRPC6 during chronic hypoxia was described to drive migration of pulmonary venous SMC in rats, whereas knockdown of TRPC6 abolishes this alteration (Wang et al., 2016). These findings could be confirmed by our study, where simultaneous deletion of TRPC1, 3 and 6 did not promote PASMC migration.

Interestingly, the finding of an unaltered pulmonary vascular muscularization between TRPC1/3/6^−/−^ and TRPC WT mice in normoxia and chronic hypoxia is in contrast to findings from single TRPC1^−/−^, TRPC6^−/−^ and double TRPC1/6^−/−^ mice, which were—at least partially—protected from CHPH, including decreased vascular remodeling (Malczyk et al., 2013; Xia et al., 2014). One might speculate that the additional deletion of the TRPC3 protein in our TRPC1/3/6^−/−^ mice causes a compensatory upregulation of other TRPC proteins, antagonizing the protective effect of the single and double knockout. One candidate for such a possible counter-regulation can be the TRPC7 protein, as the *Trpc*7 gene was found to be upregulated in hypoxia exposed PASMCs of TRPC1/3/6^−/−^ mice when compared to the hypoxic control animals (Supplementary Figure S3C).

### Loss of TRPC1, 3 and 6 alters pulmonary vasoconstriction

Since the partial protection against CHPH could not be explained by TRPC1, 3 and 6-dependent alterations in pulmonary vascular muscularization, we next investigated vascular tone and vasoreactivity to hypoxia and pharmacological stimuli by means of isolated, ventilated and perfused lungs as well as by wire myography. Our experiments indeed revealed that acute (after 10 min) and sustained hypoxia (over a timeframe of 180 min) were almost absent in TRPC1/3/6^−/−^ mice and that their vasoconstrictor response upon KCl application was reduced. Most likely the decreased RVSP in the TRPC1/3/6^−/−^ mice in chronic hypoxia is a consequence of the decreased vascular contractility as validated by the myographical data. Similar findings of less pronounced development of PH as in our study were previously published by Smith et al. (2015), where TRPC6^−/−^ mice displayed a reduced RVSP after chronic hypoxia (Smith et al., 2015). Such findings were further validated by a second study investigating TRPC1^−/−^, TRPC6^−/−^ and TRPC1/6^−/−^deficient mice, demonstrating both a reduced pulmonary arterial pressure and RVSP after chronic hypoxia (Xia et al., 2014). These changes were accompanied by decreased wall tension of pulmonary arteries isolated from chronic hypoxic TRPC1 ^−/−^ and TRPC6^−/−^ mice and are suggested to be responsible for the decreased RVSP in this study. Regarding HPV, Malczyk et al. (2013) could not detect an association between the strength of HPV and PH development in TRPC1^−/−^ mice, where RVSP was reduced after exposure to chronic hypoxia but HPV was unchanged. Thus, the reduced PH was most probably related to a decreased vascular remodeling (Malczyk et al., 2013). In contrast to this unaltered HPV in TRPC1^−/−^ mice, deletion of TRPC6 abolished the acute phase of HPV, although the sustained phase was fully developed (Weissmann et al., 2006). As already referred to above, vasoconstriction in response to both acute and sustained hypoxia was almost absent in our TRPC1/3/6^−/−^ mice. This observation might indicate that either TRPC3 alone or the combined deletion of TRPC proteins 1, 3 and 6 is responsible for this finding.

Of interest, TRPC1/3/6^−/−^ mice not only demonstrated an altered contractility of the pulmonary circulation, but of the systemic circulation as well shown by a reduced vasoconstriction of the aorta after application of KCl in normoxia compared to TRPC WT mice. However, systemic blood pressure was increased both in normoxic and hypoxic TRPC1/3/6^−/−^ mice. This discrepancy might be explained by the fact that measurements in the wire myograph do not represent the full physiological function of vessels, as e.g., the Windkessel effect of the aorta as well as confounding factors of circulating vasoactive agents are absent. Moreover, CI was unchanged between both genotypes in normoxia and hypoxia. This indicates a general effect on the systemic vasculature due to the simultaneous deletion of TRPC1, 3 and 6 rather than the occurrence of systemic hypertension or an effect due to hypoxia.

A connection between vascular tone and CHPH is not only attributed to TRPC channels, but was described for other proteins such as Rho-associated protein kinase (Fagan et al., 2004), suggesting that the partial protection against CHPH can indeed be driven by alteration in pulmonary vasoconstriction. The observed reduced vasoconstriction might be caused by a reduced [Ca^2+^]_i_ due to the simultaneous deletion of TRPC1, 3 and 6. However, since TRPC and TRPC1 channels in particular are permeable for both Ca^2+^ and Na^+^ we cannot exclude a Na^+^-mediated impact on vasoreactivity. However, it was previously demonstrated that TRPC1 is crucial for Ca^2+^ signaling and proper channel function, thus emphasizing the importance of TRPC1-dependent Ca^2+−^permeability (Dietrich et al., 2014).

In conclusion, our data provide evidence that simultaneous deletion of TRPC1, 3 and 6 partially protects against development of CHPH although vascular remodeling remains unaltered. We demonstrate that deletion of TRPC1, 3 and 6 alters (hypoxic) pulmonary vasoconstriction, which is associated with a partial protection against CHPH. The present study thus offers further evidence for TRPC1, 3 and 6 being crucially involved in the pathomechanisms of CHPH. Further studies are needed to confirm whether TRPC1, 3 and 6 might be possible treatment targets against CHPH.

## Data Availability

The original contributions presented in the study are included in the article/Supplementary Materials, further inquiries can be directed to the corresponding author.

## References

[B1] Barman Scott A. Li Xueyi Haigh Stephen Kondrikov Dmitry Mahboubi Keyvan Bordan Zsuzsanna (2019). Galectin-3 is expressed in vascular smooth muscle cells and promotes pulmonary hypertension through changes in proliferation, apoptosis, and fibrosis. Am. J. Physiol. Lung Cell. Mol. Physiol. 316 (5), L784–L797. 10.1152/ajplung.00186.2018 30724100 PMC6589585

[B2] Dietrich Alexander Fahlbusch Meike Gudermann Thomas (2014). Classical transient receptor potential 1 (TRPC1): Channel or channel regulator? Cells 3 (4), 939–962. 10.3390/cells3040939 25268281 PMC4276908

[B3] Dietrich Alexander Kalwa Hermann Rost Benjamin R. Gudermann Thomas (2005a). The diacylgylcerol-sensitive TRPC3/6/7 subfamily of cation channels: Functional characterization and physiological relevance. Pflugers Arch. 451 (1), 72–80. 10.1007/s00424-005-1460-0 15971081

[B4] Dietrich Alexander Kalwa Hermann Storch Ursula Mederos y. Schnitzler Michael Salanova Birgit (2007). Pressure-induced and store-operated cation influx in vascular smooth muscle cells is independent of TRPC1. Pflugers Arch. 455 (3), 465–477. 10.1007/s00424-007-0314-3 17647013

[B5] Dietrich Alexander Mederos y. Schnitzler Michael Gollasch Maik Gross Volkmar Storch Ursula (2005b). Increased vascular smooth muscle contractility in TRPC6-/- mice. Mol. Cell. Biol. 25 (16), 6980–6989. 10.1128/MCB.25.16.6980-6989.2005 16055711 PMC1190236

[B6] Dumitrascu Rio Weissmann Norbert Ghofrani Hossein Ardeschir Dony Eva Beuerlein Knut Schmidt Harald (2006). Activation of soluble guanylate cyclase reverses experimental pulmonary hypertension and vascular remodeling. Circulation 113 (2), 286–295. 10.1161/CIRCULATIONAHA.105.581405 16391154

[B7] Fagan Karen A. Oka Masahiko Bauer Natalie R. Gebb Sarah A. Ivy D. Dunbar Morris Kenneth G. (2004). Attenuation of acute hypoxic pulmonary vasoconstriction and hypoxic pulmonary hypertension in mice by inhibition of Rho-kinase. Am. J. Physiol. Lung Cell. Mol. Physiol. 287 (4), L656–L664. 10.1152/ajplung.00090.2003 14977625

[B8] Fernandez Ruby A. Wan Jun Song Shanshan Smith Kimberly A. Gu Yali Tauseef Mohammad (2015). Upregulated expression of STIM2, TRPC6, and Orai2 contributes to the transition of pulmonary arterial smooth muscle cells from a contractile to proliferative phenotype. Am. J. Physiol. Cell. Physiol. 308 (8), C581–C593. 10.1152/ajpcell.00202.2014 25673771 PMC4398853

[B9] Galiè Nazzareno Humbert Marc Jean-Luc Vachiery Gibbs Simon Lang Irene Torbicki Adam (2015). 2015 ESC/ERS guidelines for the diagnosis and treatment of pulmonary hypertension: The joint task force for the diagnosis and treatment of pulmonary hypertension of the European society of cardiology (ESC) and the European respiratory society (ERS): Endorsed by: Association for European paediatric and congenital cardiology (AEPC), international society for heart and lung transplantation (ISHLT). Eur. Respir. J. 46 (4), 903–975. 10.1183/13993003.01032-2015 26318161

[B10] Gierhardt Mareike Pak Oleg Sydykov Akylbek Kraut Simone Schäffer Julia Garcia Claudia (2022). Genetic deletion of p66shc and/or cyclophilin D results in decreased pulmonary vascular tone. Cardiovasc. Res. 118 (1), 305–315. 10.1093/cvr/cvaa310 33119054 PMC8752355

[B11] Grimminger Jan Richter Manuel Tello Khodr Sommer Natascha Gall Henning Ghofrani Hossein Ardeschir (2017). Thin air resulting in high pressure: Mountain sickness and hypoxia-induced pulmonary hypertension. Can. Respir. J. 2017, 8381653. 10.1155/2017/8381653 28522921 PMC5385916

[B12] Gudermann Thomas Mederos y. Schnitzler Michael Dietrich Alexander (2004). Receptor-operated cation entry--more than esoteric terminology? Sci. STKE 2004, pe35. 10.1126/stke.2432004pe35 15280577

[B13] Han MeiLan K. McLaughlin Vallerie V. Criner Gerard J. Martinez Fernando J. (2007). Pulmonary diseases and the heart. Circulation 116 (25), 2992–3005. 10.1161/CIRCULATIONAHA.106.685206 18086941

[B14] Hartmann Jana Dragicevic Elena Adelsberger Helmuth Henning Horst A. Sumser Martin Abramowitz Joel (2008). TRPC3 channels are required for synaptic transmission and motor coordination. Neuron 59 (3), 392–398. 10.1016/j.neuron.2008.06.009 18701065 PMC2643468

[B15] Hoeper Marius M. Humbert Marc Souza Rogerio Idrees Majdy Kawut Steven M. Sliwa-Hahnle Karen (2016). A global view of pulmonary hypertension. Lancet. Respir. Med. 4 (4), 306–322. 10.1016/S2213-2600(15)00543-3 26975810

[B16] Hoffman Julien I. E. (2011). Pulmonary vascular resistance and viscosity: The forgotten factor. Pediatr. Cardiol. 32 (5), 557–561. 10.1007/s00246-011-9954-3) 21432030

[B17] Jain Pritesh P. Lai Ning Xiong Mingmei Chen Jiyuan Babicheva Aleksandra Zhao Tengteng (2021). TRPC6, a therapeutic target for pulmonary hypertension. Am. J. Physiol. Lung Cell. Mol. Physiol. 321 (6), L1161–L1182. 10.1152/ajplung.00159.2021 34704831 PMC8715021

[B18] Jiang Hong-Ni Zeng Bo Chen Gui-Lan Lai Bin Lu Shao-Hua Qu Jie-Ming (2016). Lipopolysaccharide potentiates endothelin-1-induced proliferation of pulmonary arterial smooth muscle cells by upregulating TRPC channels. Biomed. Pharmacother. = Biomedecine Pharmacother. 82, 20–27. 10.1016/j.biopha.2016.04.055 27470334

[B19] Li Tangzhiming Li Suqi Feng Yilu Zeng Xiaofang Dong Shaohong Li Jianghua (2020). Combination of dichloroacetate and atorvastatin regulates excessive proliferation and oxidative stress in pulmonary arterial hypertension development via p38 signaling. Oxid. Med. Cell. Longev. 2020, 6973636. 10.1155/2020/6973636 32617141 PMC7306075

[B20] Lin Mo-Jun; Leung George P. H. Zhang Wei-Min Yang Xiao-Ru Yip Kay-Pong Tse Chung-Ming (2004). Chronic hypoxia-induced upregulation of store-operated and receptor-operated Ca2+ channels in pulmonary arterial smooth muscle cells: A novel mechanism of hypoxic pulmonary hypertension. Circ. Res. 95 (5), 496–505. 10.1161/01.RES.0000138952.16382.ad 15256480

[B21] Malczyk Monika Veith Christine Fuchs Beate Hofmann Katharina Storch Ursula Schermuly Ralph T. (2013). Classical transient receptor potential channel 1 in hypoxia-induced pulmonary hypertension. Am. J. Respir. Crit. Care Med. 188 (12), 1451–1459. 10.1164/rccm.201307-1252OC 24251695

[B22] Miller Barbara A. (2014). TRPC2. Handb. Exp. Pharmacol. 222, 53–65. 10.1007/978-3-642-54215-2_3 24756702

[B23] Pietra Giuseppe G. Capron Frederique Stewart Susan Leone Ornella Humbert Marc Robbins Ivan M. (2004). Pathologic assessment of vasculopathies in pulmonary hypertension. J. Am. Coll. Cardiol. 43, 25S–32S. 10.1016/j.jacc.2004.02.033 15194175

[B24] Pullamsetti Soni Savai Kojonazarov Baktybek Storn Samantha Gall Henning Salazar Ylia Wolf Janine (2017). Lung cancer-associated pulmonary hypertension: Role of microenvironmental inflammation based on tumor cell-immune cell cross-talk. Sci. Transl. Med. 9 (416), eaai9048. 10.1126/scitranslmed.aai9048 29141888

[B25] Ryan John J. Archer Stephen L. (2014). The right ventricle in pulmonary arterial hypertension: Disorders of metabolism, angiogenesis and adrenergic signaling in right ventricular failure. Circ. Res. 115 (1), 176–188. 10.1161/CIRCRESAHA.113.301129 24951766 PMC4112290

[B26] Sato Atsushi Okada Masashi Shibuya Keita Watanabe Eriko Seino Shizuka Narita Yoshitaka (2014). Pivotal role for ROS activation of p38 MAPK in the control of differentiation and tumor-initiating capacity of glioma-initiating cells. Stem Cell. Res. 12 (1), 119–131. 10.1016/j.scr.2013.09.012 24185179

[B27] Schermuly Ralph T. Ghofrani Hossein A. Wilkins Martin R. Grimminger Friedrich (2011). Mechanisms of disease: Pulmonary arterial hypertension. Nat. Rev. Cardiol. 8 (8), 443–455. 10.1038/nrcardio.2011.87 21691314 PMC7097518

[B28] Seimetz Michael Parajuli Nirmal Pichl Alexandra Veit Florian Kwapiszewska Grazyna Weisel Friederike C. (2011). Inducible NOS inhibition reverses tobacco-smoke-induced emphysema and pulmonary hypertension in mice. Cell. 147 (2), 293–305. 10.1016/j.cell.2011.08.035 22000010

[B29] Smith Kimberly A. Ayon Ramon J. Tang Haiyang Makino Ayako Yuan Jason X-J. (2016). Calcium-Sensing receptor regulates cytosolic Ca 2+ and plays a major role in the development of pulmonary hypertension. Front. Physiol. 7, 517. 10.3389/fphys.2016.00517 27867361 PMC5095111

[B30] Smith Kimberly A. Voiriot Guillaume Tang Haiyang Fraidenburg Dustin R. Song Shanshan Yamamura Hisao (2015). Notch activation of Ca(2+) signaling in the development of hypoxic pulmonary vasoconstriction and pulmonary hypertension. Am. J. Respir. Cell. Mol. Biol. 53 (3), 355–367. 10.1165/rcmb.2014-0235OC 25569851 PMC4566064

[B31] Sommer Natascha Alebrahimdehkordi Nasim Pak Oleg Knoepp Fenja Strielkov Ievgen Scheibe Susan (2020). Bypassing mitochondrial complex III using alternative oxidase inhibits acute pulmonary oxygen sensing. Sci. Adv. 6 (16), eaba0694. 10.1126/sciadv.aba0694 32426457 PMC7159913

[B32] Sommer Natascha Hüttemann Maik Pak Oleg Scheibe Susan Knoepp Fenja Sinkler Christopher (2017). Mitochondrial complex IV subunit 4 isoform 2 is essential for acute pulmonary oxygen sensing. Circ. Res. 121 (4), 424–438. 10.1161/CIRCRESAHA.116.310482 28620066 PMC5544581

[B33] Stenmark Kurt R. Frid Maria G. Graham Brian B. Tuder Rubin M. (2018). Dynamic and diverse changes in the functional properties of vascular smooth muscle cells in pulmonary hypertension. Cardiovasc. Res. 114 (4), 551–564. 10.1093/cvr/cvy004 29385432 PMC5852549

[B34] Stenmark Kurt R. McMurtry Ivan F. (2005). Vascular remodeling versus vasoconstriction in chronic hypoxic pulmonary hypertension: A time for reappraisal? Circ. Res. 97 (2), 95–98. 10.1161/01.RES.00000175934.68087.29 16037575

[B35] Stenmark Kurt R. Meyrick Barbara Galie Nazzareno Mooi Wolter J. McMurtry Ivan F. (2009). Animal models of pulmonary arterial hypertension: The hope for etiological discovery and pharmacological cure. Am. J. Physiol. Lung Cell. Mol. Physiol. 297 (6), L1013–L1032. 10.1152/ajplung.00217.2009 19748998

[B36] Tajsic Tamara Morrell Nicholas W. (2011). Smooth muscle cell hypertrophy, proliferation, migration and apoptosis in pulmonary hypertension. Compr. Physiol. 1 (1), 295–317. 10.1002/cphy.c100026 23737174

[B37] Tang Haiyang Yamamura Aya Yamamura Hisao Song Shanshan Fraidenburg Dustin R. Chen Jiwang (2016). Pathogenic role of calcium-sensing receptors in the development and progression of pulmonary hypertension. Am. J. Physiol. Lung Cell. Mol. Physiol. 310 (9), L846–L859. 10.1152/ajplung.00050.2016 26968768 PMC4867349

[B38] Tuder Rubin M. Marecki John C. Richter Amy Fijalkowska Iwona Flores Sonia (2007). Pathology of pulmonary hypertension. Clin. Chest Med. 28 (1), 23–42. 10.1016/j.ccm.2006.11.010 17338926 PMC1924722

[B39] Veith Christine Vartürk-Özcan Ipek Wujak Magdalena Hadzic Stefan Wu Cheng-Yu Knoepp Fenja (2022). SPARC, a novel regulator of vascular cell function in pulmonary hypertension. Circulation 145 (12), 916–933. 10.1161/CIRCULATIONAHA.121.057001 35175782

[B40] Vonk Noordegraaf A. Galiè N. (2011). The role of the right ventricle in pulmonary arterial hypertension. Eur. Respir. Rev. 20 (122), 243–253. 10.1183/09059180.00006511 22130817 PMC9487747

[B41] Wang Jian Jiang Qian Wan Limei Yang Kai Zhang Yi Chen Yuqin (2013). Sodium tanshinone IIA sulfonate inhibits canonical transient receptor potential expression in pulmonary arterial smooth muscle from pulmonary hypertensive rats. Am. J. Respir. Cell. Mol. Biol. 48 (1), 125–134. 10.1165/rcmb.2012-0071OC 23065131 PMC3547081

[B42] Wang Qingjie Wang Dong Yan Gaoling Sun Ling Tang Chengchun (2016). TRPC6 is required for hypoxia-induced basal intracellular calcium concentration elevation, and for the proliferation and migration of rat distal pulmonary venous smooth muscle cells. Mol. Med. Rep. 13 (2), 1577–1585. 10.3892/mmr.2015.4750 26718737 PMC4732854

[B43] Weissmann Norbert Dietrich Alexander Fuchs Beate Kalwa Hermann Ay Mahmut Dumitrascu Rio (2006). Classical transient receptor potential channel 6 (TRPC6) is essential for hypoxic pulmonary vasoconstriction and alveolar gas exchange. Proc. Natl. Acad. Sci. U. S. A. 103 (50), 19093–19098. 10.1073/pnas.0606728103 17142322 PMC1748182

[B44] Weissmann Norbert Manz Daniel Buchspies Daniela Keller Stephan Mehling Tanja Voswinckel Robert (2005). Congenital erythropoietin over-expression causes "anti-pulmonary hypertensive" structural and functional changes in mice, both in normoxia and hypoxia. Thromb. Haemost. 94 (3), 630–638. 10.1160/TH05-02-0104 16268482

[B45] Wijeratne D. Thiwanka Lajkosz Katherine Brogly Susan B. Lougheed M. Diane Jiang Li Housin Ahmad (2018). Increasing incidence and prevalence of world health organization groups 1 to 4 pulmonary hypertension: A population-based cohort study in ontario, Canada. Circ. Cardiovasc. Qual. Outcomes 11 (2), e003973. 10.1161/CIRCOUTCOMES.117.003973 29444925 PMC5819352

[B46] Xia Yang Yang Xiao-Ru Fu Zhenzhen Paudel Omkar Abramowitz Joel Birnbaumer Lutz (2014). Classical transient receptor potential 1 and 6 contribute to hypoxic pulmonary hypertension through differential regulation of pulmonary vascular functions. Hypertension 63 (1), 173–180. 10.1161/HYPERTENSIONAHA.113.01902 24144647 PMC4102175

[B47] Xu Juan Wen Xing Fu Zhenli Jiang Yongliang Hong Wei Liu Rongmin (2021). Chronic hypoxia promoted pulmonary arterial smooth muscle cells proliferation through upregulated calcium-sensing receptorcanonical transient receptor potential 1/6 pathway. Microcirculation 28 (6), e12715. 10.1111/micc.12715 34008915

[B48] Yu Ying Fantozzi Ivana Remillard Carmelle V. Landsberg Judd W. Kunichika Naomi Platoshyn Oleksandr (2004). Enhanced expression of transient receptor potential channels in idiopathic pulmonary arterial hypertension. Proc. Natl. Acad. Sci. U. S. A. 101 (38), 13861–13866. 10.1073/pnas.0405908101 15358862 PMC518765

[B49] Yue Yun Li Yi-Qi Fu Shu Wu Yu-Ting Zhu Ling Hua Liang (2020). Osthole inhibits cell proliferation by regulating the TGF-β1/Smad/p38 signaling pathways in pulmonary arterial smooth muscle cells. Biomed. Pharmacother. = Biomedecine Pharmacother. 121, 109640. 10.1016/j.biopha.2019.109640 31810114

