# Supplementary figures and images for: Deletion of classical transient receptor potential 1, 3 and 6 alters pulmonary vasoconstriction in chronic hypoxia-induced pulmonary hypertension in mice
Source: Front Physiol. 2022 Dec 7;13:1080875. doi: 10.3389/fphys.2022.1080875 (PMC9768328; doi:10.3389/fphys.2022.1080875)

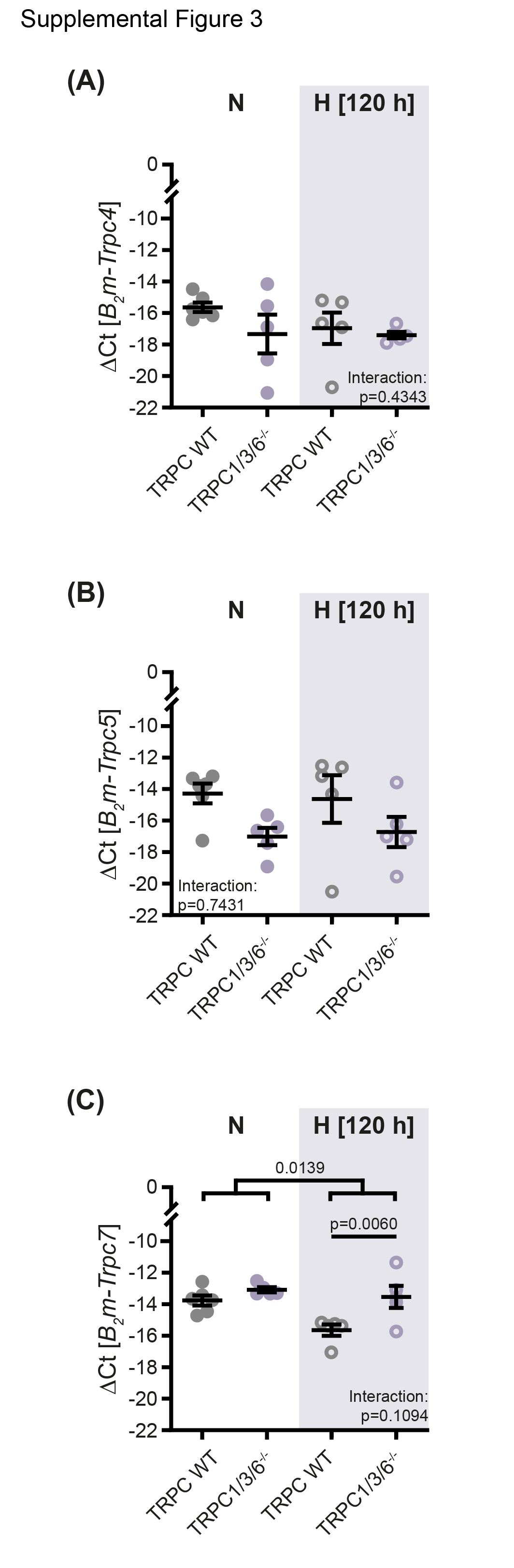

Supplement: Supplementary file 1 [file Image3.jpeg]

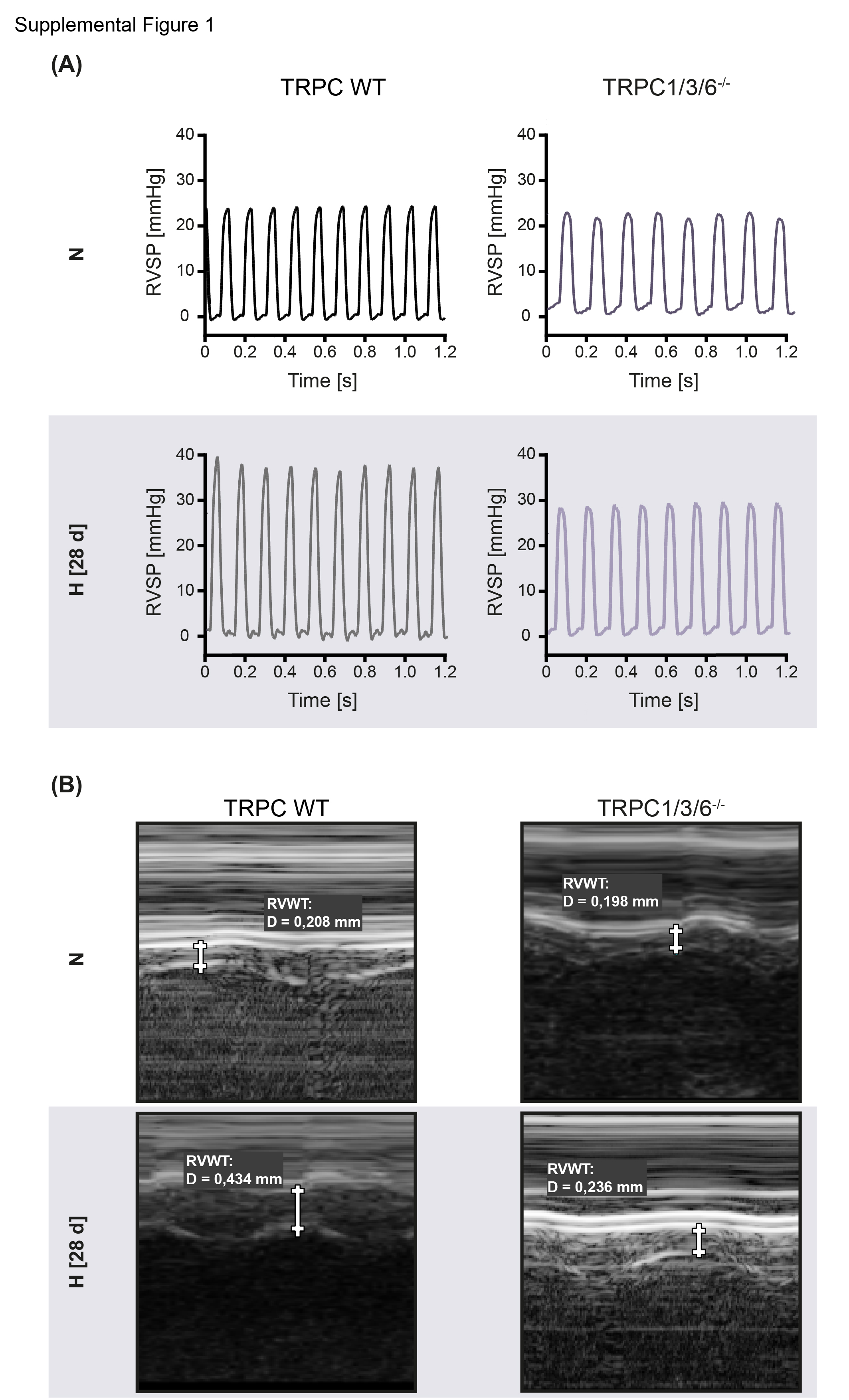

Supplement: Supplementary file 2 [file Image1.jpeg]

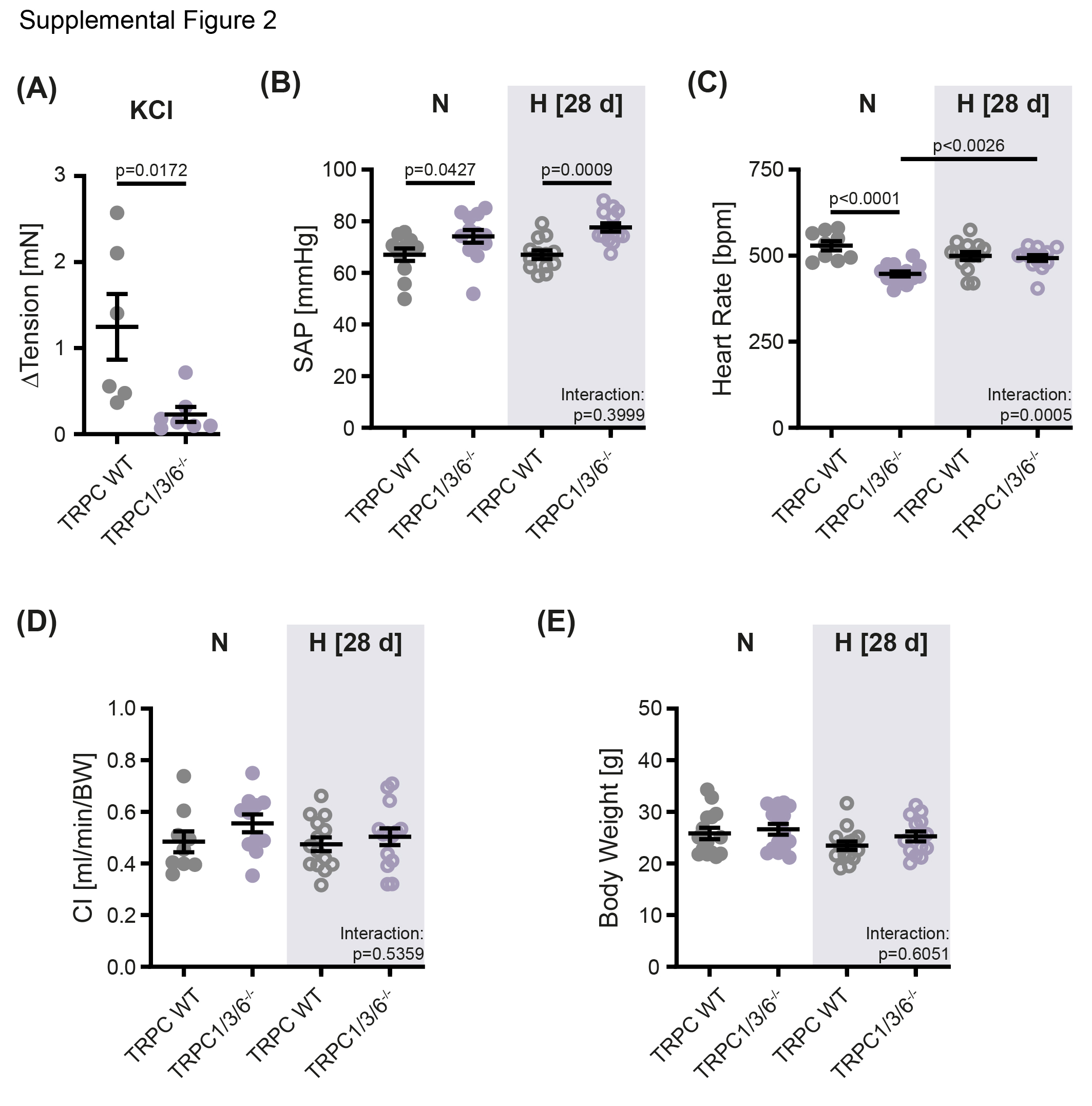

Supplement: Supplementary file 3 [file Image2.jpeg]
